# Perinatal social work during the Covid-19 pandemic: Reflecting on concepts of time and liminality

**DOI:** 10.1177/1473325020973306

**Published:** 2021-03

**Authors:** Elaine Wilson, Kaylene Jackson, Aoife Shannon

**Affiliations:** School of Social Policy, Social Work and Social Justice, 8797 University College Dublin, Dublin, Ireland; 58003 National Maternity Hospital, Dublin, Ireland

**Keywords:** Health, practice research, reflective practice, perinatal social work

## Abstract

This article reflects upon the experiences of two perinatal, hospital social workers during the unprecedented time of the Covid-19 in Ireland, as discussed with their academic colleague. This encounter revealed the complexity of service delivery that emerged, when managing the needs of vulnerable clients whilst being mindful of personal safety. One of the social workers was pregnant so was conscious of possible risks to her unborn child, as well as her young family at home. The second social worker, her line manager, discusses the dilemmas associated with the management of risk when allocating staff to contexts where they would be in direct contact with Covid-19. At the core of the analysis of these situations is the notion of liminal space and the realisation that time appears to have a new meaning; what we once knew as normal no longer exists, but we have yet to reach the ‘new normal’.

## Introduction

This article reflects upon the experiences of two perinatal, hospital social workers during the unprecedented time of the Covid-19 in Ireland, as discussed with their academic colleague. This encounter revealed the complexity of service delivery that emerged, when managing the needs of vulnerable clients, whilst being mindful of personal safety. One of the social workers was pregnant so was conscious of possible risks to her unborn child, as well as her young family at home. The second social worker, her line manager, discusses the dilemmas associated with the management of risk when allocating staff to contexts where they would be in direct contact with Covid-19. At the core of the analysis of these situations is the notion of liminal space and the realisation that time appears to have a new meaning; what we once knew as normal no longer exists, but we have yet to reach the ‘new normal’.

## Background

 Perinatal social work in Ireland is informed by the National Maternity Strategy (2016–2026) (Department of Health, 2016), a 10-year strategic plan which emphasises the need for women-centred approaches and the assertion that pregnancy and childbirth are normal life stages. It has become apparent that Covid-19 has seriously challenged these aspirations by disrupting opportunities for normalising experiences of pregnancy and childbirth. The conventional supports that enable women to have good experiences of hospital attendance for antenatal visits, ultrasound scans, inpatient admissions, induction of labour and neonatal care have been eroded. Thus, a support person can only be present in the labour ward or in theatre for a caesarean section. Initially visits would between only fifteen and thirty minutes per day, although these restrictions have gradually been relaxed, post-lockdown to become between one hour and two hours per day.

These significant changes bring with them a myriad of smaller, sometimes, hidden problems and challenges which can adversely affect the pregnancy journey, including:

- Women receiving bad news without family and carers being present
- Women experiencing adverse pregnancy events/outcomes alone
- Fathers missing and excluded from key events which may impact on future bonding and emotional adjustment for children and parents
- Increased possibilities of psychological impact caused by separation from sick or preterm infants

## The impact on social work and the professional response

Social workers, historically, play key roles in such services and, as with other health care professionals, have been adversely affected during previous epidemics. Rowlands (2007) describes how, during the SARS epidemic in Singapore, they experienced fears of infection, curtailment of certain liberties, and concerns for their own family members health (2007: 59). This section now discusses how the two Irish social workers dealt with Covid-19 issues in their practice.

Shannon, senior social worker, discussed her anxieties around the nexus between professional and personal lives: “*At the start of my own pregnancy the world was still as I knew it, I waited impatiently to get to the twelve-week mark where I could hope risks of pregnancy loss would be reduced and news could be shared with families and friends. However, life has since changed dramatically. It has been challenging working with antenatal and postnatal patients whose fears of the Covid-19 situation mirror that of my own”*. She struggled to find the balance between supporting women and families and dealing with these anxieties. Whilst striving to provide a client centred approach and supporting women, for example, through distress and trauma of having their baby admitted to a Neonatal Intensive Care, it was not possible to disregard her own feelings of risk to her unborn child in the midst of Covid-19. As she reflected, *“I feel I am an active living part of the evidence being gathered and experience of what it is to be pregnant and a health care worker”.* Pragmatic approaches to service delivery were developed yet many dilemmas remained. As she put it: *PPE helps to provide a barrier to transmission but also provides barriers to how we communicate, the message we are sending to families that we or they pose threats and is it actually effective or necessary? Mixed messages appear to exist, and countries globally appear to have different advice on even mask wearing’.*

Jackson, the head medical social worker, struggled to deliver the social work service which was governed by the 2 m/15-minute rule used to minimise infections and issues of risk and risk management were constantly intruding on decision-making. She characterised her management responsibilities before Covid-19 in terms of a *“a dichotomous pursuit where the work needs to be done well and the interests of vulnerable service users met while ensuring that staff are protected from stress, vicarious trauma and burnout. For the most part these can be met alongside each other”.* However, Covid-19 has brought these two areas into an uncomfortable tension. Staff are being asked to provide the same service to vulnerable service users when perceptions about their own wellbeing (physical, emotional, and psychological) were ever present. It materialised that managing these competing priorities could be overwhelming and required new management strategies and priorities. As at other moments of past crisis, although there had never been anything as potentially catastrophic as Covid-19, there were painful realisations that mistakes were being made and “*judgements clouded in unchartered territory”*. Although it was possible to provide staff with the physical components of protection such as masks, visors, and scrubs, it is far more difficult to ensure that staff receive the emotional and physical support they needed in the midst of the pandemic. An example of this was when a staff member was allocated work with a woman in the hospital’s neonatal intensive care unit which normally required both personal touch and time, a major asset of the social work service. Both Jackson and Shannon have become increasingly concerned about the erosion of this service and the impact upon clients. Complex assessments of need and risk are hampered by the limitations of phone-based discussions and the lack of face to face contact with vulnerable patients and families. The palpable dilemma for Jackson was to do her best in maintaining the quality of the services, whilst balancing a duty of care for her staff. These issues were magnified within the small social work team where there were variable levels of tolerance and personal comfort in in dealing with, and being confronted by, Covid -19 related issues. Jackson must ensure that there are equitable workloads and systems of support to enable *“staff to manage feelings of fear for their own safety, as well as impotence and helplessness about their work”.* This has now become an essential component of the management role, whilst also continuing with the everyday tasks of service planning and delivery.

## Discussion

The concept of time was constantly raised by the social workers in these discussions. On the one hand time had appeared to become almost meaningless because Covid-19 had profoundly disrupted the ‘normal’ rhythms of everyday policy and practice. It was, and continues to be unclear, about when or if the restrictions would be lifted completely. The social workers found that time had become far more defined and prescriptive, essentially determined by the strictures of risk management and protocol. The notion of liminal space is also helpful in analysing the impact of Covid-19 on the life of patients and staff. A liminal space refers to an in-between space and is often used to describe a situation where someone is on the verge of something new. It is often an uncomfortable place to be because you have moved from what you once knew but have not yet reached your destination. Wilson (2019) utilised this concept in her work exploring the transition from treatment to survivorship with early stage breast cancer patients. An example of one of the difficulties faced in this liminal space was that the women no longer knew how to define themselves – were they still cancer patients, or should they say they had had cancer. For them time was also very prominent in their minds, with five years without recurrence being a very significant milestone to aim for. Although the women in Wilson’s study found the transition difficult at times, there was also the sense that this liminal space was also a transformative stage, offering the opportunity for reflection and growth. The pandemic has ensured that our understanding of life and death has been adjusted and reconsidered as restrictions and responses continually shift. This is a liminal space which continues, with no end in sight where we have moved from what we expected to be normal into a type of interim world, characterised by Turner using the binary ‘betwixt and between’ (1969). Persistent uncertainties remain about how long restrictive measures would be in place and the way in which vigilance heightens the sense of loss of control over one’s own life, and how much one’s life is in the control of another.

As a pregnant healthcare worker Shannon reflected on how time as a concept has never seemed more fluid to her and yet the time restrictions caused by the 15 minute protocol were a constant reminder of the presence of the pandemic leading to a constant, never ending, systems of brief reviews. Then this sense of contradiction and anxiety becomes translated to her own personal experiences: “*in addition to this fluidity and rigidity is the very definite time frame of a pregnancy episode, or my goal, which is to get to 28 weeks after which time my way of working will again need to be revised, reviewed and refreshed. The selfish part of me being somewhat relieved that seeing patients will be taken out of my hands while the guilt of not being an active member of my team will still be ever present”.*

What became apparent through the conversations with both perinatal social workers was the intersection between time and risk. Almost all social workers had encountered situations where they have had to weigh up the risk of working with a highly emotional or aggressive client. The difference between that and the Covid-19 situation is that weighing up the risk during this pandemic involves weighing up the potential risk of bringing the disease home to family and friends. Although it is easy to view the impact of Covid-19 as wholly negative, Jackson believes that it has brought with it some positives: *“The goal is to allow the crisis provide an opportunity to explore and develop the new. Reviewing even the most concrete practices within the Social Work Department has become normative”.* For example, the impact of Covid-19 had resulted in a fresh and energised focus on services for those who suffer domestic violence and abuse. Reduced footfall due to visitor restrictions has created opportunities for ward based ad hoc training and updated patient information packs and awareness campaigns have been launched with a greater social media presence, as patient care has shifted on-line. Individual presentations in the emergency ward, previously lost in the crowd, are now become more visible, creating opportunities for managing issues of risk and need. The necessary management and reimagining of time has led to new forms of working where there are new shift and work patterns to enable necessary social distancing. The two social work teams practice using three 12-hour shifts resulting in a renewed energy and better management of work/life balance.

## Contemplating the future

It is essential that we reflect upon how Covid-19 has impacted upon our personal and professional lives and the social work response. The anxieties expressed by Shannon and Jackson in the particular contexts of maternity and neo-natal services one assumes are played in other social work services. It is therefore crucial that the profession finds way to mediate the adverse effects of the pandemic and to recalibrate our understanding of practice modalities. For example, the barriers created by Covid-19 can be interrupt opportunities to build therapeutic relationships in health care settings as described earlier. Despite these problems, new spaces emerge for positive change and transformation. We also need to find new ways of managing threats to our health and well-being. In reflecting upon her experiences, Shannon recognised the fears expressed by her clients as fears that she herself was experiencing for herself and her unborn child. Such self-reflection can be protective. Raven et al. (2018) examined health care professionals’ experiences of working in Sierra Leone during the Ebola Virus epidemic and found considerable reserves of resilience amongst this population. They argued that such forms of resilience can be nurtured and reinforced through supervision, peer support networks and better use of communication technologies.

As we find ways of reflecting upon what Covid-19 means for society, family, and social work practice we must open up to the ambiguities and contradictions that are both challenging but also create opportunities for change. In the midst of these difficulties and the uncharted waters we are navigating, such situations of crisis create the potential for growth and invention. The social work profession plays a key role in many health care systems so these and voices such as those of Shannon and Jackson will enable new forms of service delivery to develop. The challenge is building these coping mechanisms into routine systems, pre-empting shocks, rather than waiting to respond belatedly to crises’ (Raven et al., 2018).
